# MEIoT 2D-CACSET: IoT Two-Dimensional Cartesian Coordinate System Educational Toolkit Align with Educational Mechatronics Framework

**DOI:** 10.3390/s22134802

**Published:** 2022-06-25

**Authors:** Rocío Carrasco-Navarro, Luis F. Luque-Vega, Jesús Antonio Nava-Pintor, Héctor A. Guerrero-Osuna, Miriam A. Carlos-Mancilla, Celina Lizeth Castañeda-Miranda

**Affiliations:** 1 Research Laboratory on Optimal Design, Devices and Advanced Materials—OPTIMA, Department of Mathematics and Physics, ITESO, Tlaquepaque 45604, Jalisco, Mexico; rociocarrasco@iteso.mx; 2 Centro de Investigación, Innovación y Desarrollo Tecnológico CIIDETEC-UVM, Universidad del Valle de México, Tlaquepaque 45601, Jalisco, Mexico; miriam_carlos@my.uvm.edu.mx; 3 Posgrado en Ingeniería y Tecnología Aplicada, Universidad Autónoma de Zacatecas, Zacatecas City 98000, Zacatecas, Mexico; jesus.nava@uaz.edu.mx (J.A.N.-P.); hectorguerreroo@uaz.edu.mx (H.A.G.-O.); celina@uaz.edu.mx (C.L.C.-M.); 4 Unidad Académica de Ingeniería Eléctrica, Universidad Autónoma de Zacatecas, Zacatecas City 98000, Zacatecas, Mexico

**Keywords:** Internet of Things, sensors, engineering education, educational mechatronics, educational toolkit

## Abstract

The educational sector has made extraordinary efforts to neutralize the impact of the pandemic caused by COVID-19, forcing teachers, scholars, and academic personnel to change the way education is delivered by developing creative and technological solutions to improve the landscape for education. The Internet of Things (IoT) is crucial for the educational transition to digital and virtual environments. This paper presents the integration of IoT technology in the Two-Dimensional Cartesian Coordinate System Educational Toolkit (2D-CACSET), to transform it into MEIoT 2D-CACSET; which includes educational mechatronics and the IoT. The Educational Mechatronics Conceptual Framework (EMCF) is extended to consider the virtual environment, enabling knowledge construction in virtual concrete, virtual graphic, and virtual abstract levels. Hence, the students acquire this knowledge from a remote location to apply it further down their career path. Three instructional designs are designed for this work using the MEIoT 2D-CACSET to learn about coordinate axes, quadrants, and a point in the 2D Coordinate Cartesian System. This work is intended to provide an IoT educational technology to offer an adequate response to the educational system’s current context.

## 1. Introduction

The educational sector has suffered considerable disruption because of COVID-19, officially declared by the World Health Organization on 11 March 2020 [1]. It has affected the academic performance of undergraduate students in many fields of knowledge [2]. Since then, teachers, scholars, and educational personnel have tried to neutralize the impact of the pandemic that forced them to change the way education is delivered by developing creative and technological solutions to improve the landscape for education; however, new challenges have emerged. Several works [3,4,5] emphasize the challenge of transferring to a remote version of the classes focused on practical experience, instructing future engineers to operate industrial/specialized equipment that is often not easily accessible to a student without a university lab or similar. The preceding has much more relevance with the arrival of Industry 4.0, which requires specialization of students according to the critical technologies of the new industry generation. The areas include autonomous robots, the Internet of Things, additive manufacturing, augmented reality, cybersecurity, cloud computing, big data, and data analysis, thus creating a gap even wider between academia and industry [6].

Several works propose different alternatives to mitigate this situation; an example is the employment of remote laboratories, which are defined as an interface that allows performing experiments that can be used and controlled by students online without direct contact with the equipment used [7]. Some works have been proposed before COVID-19 but were never fully realized and had to change by the pandemic. In [8], authors offer the development of a remote laboratory called RLAB-UOC for engineering education focused on teaching telecommunications engineering, which uses specialized hardware: FPGA, NI ELVIS II, and USRP-2920 and software: LabVIEW, that allows students to perform practices related to electronics, communications, and radio-communications, the laboratory is entirely online and can be accessed by students anywhere and anytime.

In [9], the Lab-Tech@Home project is implemented and attempts to help understand topics related to control engineering. Using open-source software, students feel motivated as they can practice from a remote classroom. In [10], the authors survey the impact of virtual and remote manufacturing laboratories focused on teaching material characterization at the Technical University of Dortmund. The lab involves robotics machines, drawing testing machines, and Zwick machines. Students have multiple options; they can access the virtual twin environment of the remote laboratory to familiarize themselves with actual equipment. Instructors use live-stream cameras to demonstrate practices in real time; students can interact and select different camera perspectives. Several works involve virtual reality (VR) [11], the use of motor current signature analysis (MCSA) to detect induction motor failures, and augmented reality (AR) [12], where AR is applied to teaching architecture, engineering construction subjects, and Internet of Things (IoT). All agree that employing these technologies in educational applications increases students’ interest, helps them acquire new skills through accomplishments, and enables interaction with remote experts. Additionally, beyond promoting solutions suitable for the COVID era, these works are closely related to Industry 4.0. They are directed towards education 4.0: where learning is based mainly on active learning, online distance learning leverages technological platforms to enact remote processes applying digitalization, virtualization, and connectivity through interactive and hands-on learning activities that immerse students in flexible digital models employing new and different strategies and pedagogical procedures for the teaching–learning process [13,14] that will be very important in a post-COVID era [15].

The main contribution of this work is focused on the adaptation of an educational kit, conceived for a face-to-face education with teacher and physical attendance of the students in the classroom, to an smart educational kit that takes advantage of the IoT technology. IoT can drive the transformation to turn physical laboratories into online remote laboratories connected to the internet. A complete and flexible IoT setup solution was developed to transform the Two-Dimensional Cartesian Coordinates Toolkit (2D-CACSET) [16] into the Educational Mechatronics (ME for its acronym in Spanish) and the Internet of Things 2D-CACSET (MEIoT-2D-CACSET), allowing a remote hands-on-learning (HOL) laboratory. It is flexible since different educational kits can be transformed into smart educational kits, allowing new online remote scenarios for more advanced topics and/or interaction methods. In particular, in the scenario described in this paper, the participant can manipulate the mobile robot by remotely commanding it through the GUI. Moreover, this work has led to the improvement and extension of the EMCF by including the “virtual” macro process learning that uses interconnected devices to give the complete experience of each learning level to the participant in the GUI. This new learning environment aims to progressively expand the students’ learning methods and learning environment resources.

The motivation behind the work presented in this article is to enable students to apply the Educational Mechatronics Conceptual Framework (EMCF) methodology from a remote location to gain knowledge and proficiency in the scope of spatial thinking, which is crucial for success in engineering [17]. The paper is detailed as follows: Section 2 presents some related works; Section 3 describes the educational mechatronics conceptual framework applied in a Cartesian plane and the development of mechatronic thinking. Section 4 presents the design and implementation of the MEIoT-2DCACSET using the IoT National Digital Observatory of Smart Environments (OBNiSE by its initials in Spanish) architecture; Section 5 details the implementation of the MEIoT-2D-CACSET and the interaction between its elements. In Section 6, the instructional design for the METoT-2DCACSET is described. The model is divided into three levels: concrete, graphic, and abstract, in which different practices are implemented. Finally, a discussion is given in Section 7.

## 2. Related Work

IoT is a paradigm that provides the instruments needed to enable a global network of interconnected heterogeneous elements (things) that extract, create, and contextualize information according to the objective. IoT allows the evolution of traditional communications into a scenario where communications can be between human–things, things–human, or a machine-to-machine (M2M) [18]. Moreover, IoT is one of the leading technologies of Industry 4.0; therefore, to provide qualified professionals, IoT should be part of academia as a study subject and as part of how education is delivered [19,20,21]. These works highlight the value of educational growth and development of high-tech skills, focusing on digital technologies, and replacing traditional modes of education. Individual and self-learning models empower the student thanks to digital technologies. However, they must still develop the skills and abilities to manage new technological devices.

In this context, this section reviews some works that employ IoT to develop teaching–learning solutions to give us different valuable perspectives for our proposal.

It is worth mentioning that since 2D-CACSET is an instructional face-to-face learning tool, papers that provide IoT capabilities to non-internet-capable devices or develop remote laboratories oriented to teach specific subjects are of interest to our purpose. A case in point is [22], where an IoT field programmable gate array (FPGA) remote laboratory is presented and oriented to a university-level digital design course; authors employ single board computers (SBC) to provide IoT capabilities to FPGA. The laboratory involves a mobile application and two FPGA-based development boards. The mobile application displays the pin state of FPGA in real time and allows programming remotely, permitting multiple users to collaborate. The laboratory is scalable to add more FPGA development boards and will help mitigate distance learning issues.

The previous provides IoT capabilities and enables hardware to be remotely accessed by students; however, other works, such as [23], propose enabling students’ hardware to be accessed by the teacher. The solution is oriented to teach the use of microcontrollers. The authors create an IoT system that provides a network connection between the teacher and the students using an IoT monitoring device. The system includes internet capabilities to an STM32 development board; the IoT system allows sending of information about the microcontroller program the student is working on and provides control of the complete hardware; this makes it possible to control and watch the student activity during the lesson.

Similarly, in [24], the authors develop a cloud-based laboratory for teaching electronic subjects. This lab involves infrared ambient light and temperature sensors, an integrated National Instruments myRIO (NI myRIO) device, and the Laboratory Virtual Instrumentation Engineering Workbench (LabVIEW) software, in conjunction with NI SystemLink Cloud, to offer a learning platform for instructors and students. A computer provides IoT that allows reconfiguring the platform according to the learning topic. Thus, students perform different experiments and interact with the instructor.

Some works have mentioned that the IoT has been applied in subjects related to wireless networks or specialized hardware; however, many other areas of knowledge use IoT. The work presented in [25] proposes a methodology to support teaching in biomedical engineering based on cloud computing and IoT technology. An MKR WiFi 1010 baseboard is connected to an IoT cloud service Google Science Journal and registers the activity of several biomedic sensors; electrocardiography, electromyography, electroencephalography, and electrodermal activity. Through Jupiter Notebooks, students can develop scripts to process and extract analytics. The authors in [26] create a virtual remote laboratory (ViRe-Lab) that employs virtualization technologies for cybersecurity in information systems; this laboratory emulates realistic scenarios by applying practical activities where students learn to detect vulnerabilities in a network system, report them, and solve. Those activities allow acquiring a set of skills for implementation over IoT devices.

These works have shown that the synergy of education and new technologies, such as IoT, lead to more efficient learning, removing geographic location barriers, language, and economics [27]. Moreover, this synergy promotes the idea that students cease being simple knowledge receivers of information and transforms them to become applicators thanks to active learning and the remote interactions allowed by technology.

## 3. Educational Mechatronics Conceptual Framework EMCF for Virtual Scenarios

**Figure 1:**
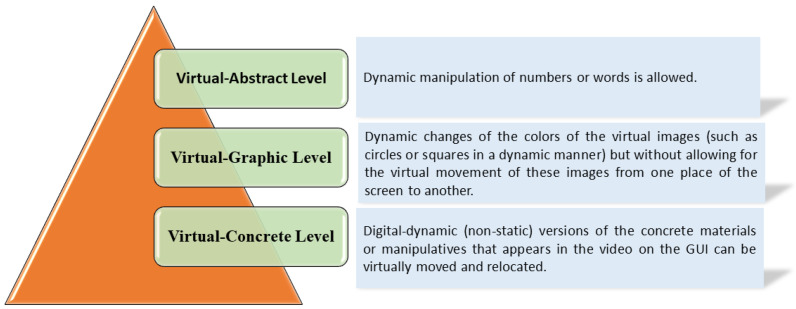
Level of interaction with the MEIoT components used in the process from a digital and dynamic perspective.

Since this project aims to remove the barrier of physical location and geography, the EMCF Learning Construction Methodology (EMCF-LCM) presented in [28,29] is extended to include virtual levels involving an interactive, technology-enabled remote educational toolkit with a dynamic and remote object. These include all the programmable features that allow it to be manipulated for students in real time, which presents opportunities for constructing knowledge. For this reason, in this work, “virtual” includes both digital and dynamic components (with the possibility of virtual movement and manipulation from the graphical user interface (GUI)). The virtual level is considered to have three sub-levels: virtual-concrete, virtual-pictorial, and virtual-abstract (see Figure 1).

The virtual-concrete level: this process implies that digital-dynamic (non-static) versions of the concrete materials or manipulatives in the video on the GUI can be virtually moved and relocated. The virtual-pictorial level allows dynamic color changes of the virtual images (such as circles or squares in a diligent manner) but without allowing for the virtual movement of these images from one place on the screen to another. The virtual-abstract level allows for the dynamic manipulation of numbers or words [30].

The virtual-levels involve interactive technology that enables visual interaction of a dynamic remote object, including all of the features that allow it to be manipulated in a graphical user interface. This presents the opportunity for constructing knowledge. It can be noted that the object also can be just a representation of a dynamic mathematical object [31].

## 4. Design and Implementation of the MEIoT 2D-CACSET in the OBNiSE Architecture

Nowadays, IoT is one of the leading technologies required by industry and education. Especially with the epidemic, virtual education has become essential. It has allowed students to adapt to circumstances to develop and implement virtual prototypes using IoT strategies, data analysis, and artificial intelligence, among others.

These IoT strategies have been supported by institutional education responsible for adapting and creating new techniques, tools, and methodologies for students to use everywhere, anytime. For instance, the kit of educational technology developed in this paper uses the platform of the OBNiSE architecture [32]. This architecture allows adaptation, creation, monitoring, and development of IoT solutions for smart cities, healthcare, mobility, technology, and education.

The architecture comprises six layers, including device connections, network, processing, security, cloud, and applications, as described below.

- The application and device layers allow the integration of several solutions from different areas to propose comprehensive solutions in education, smart cities, and healthcare, among others. The educational mechatronics application for this paper describes the macro-process of the EMCF-LCM presented in Figure 1, integrated with the ONBiSE architecture.
- The network layers allow to connect people to the network and set profiles, permissions, and availability of services. The configuration of this layer is through tools, user profiles, and data accessibility.
- The processing layer is responsible for processing the information and organizing the data to be visualized.
- The cloud layer ensures the data availability of users, systems, and applications.
- Finally, the security layer manages the data encryption of all the layers and secures the information in the system.

**Figure 2:**
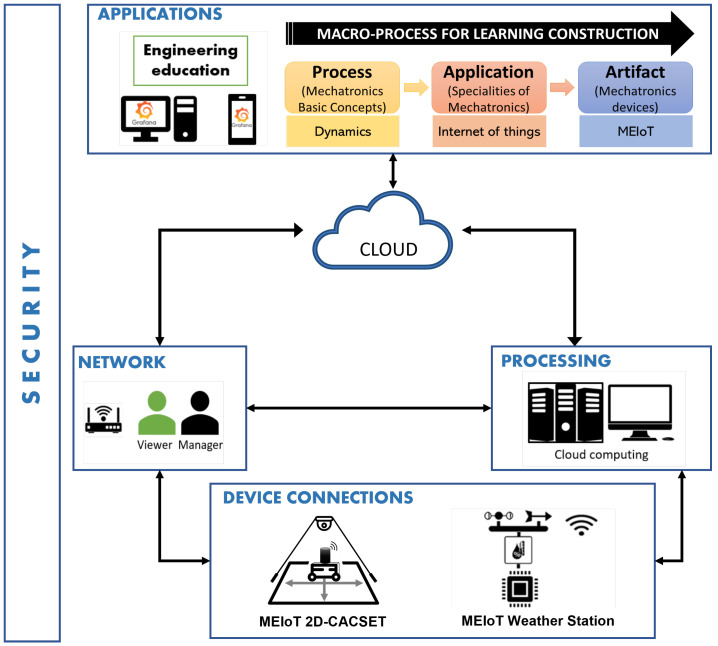
The OBNiSE architecture for the design and implementations of IoT solutions.

Currently, the OBNiSE architecture platform has two functional educational devices: (1) the MEIoT weather station proposed by [33], and (2) MEIoT 2D-CACSET, detailed in this paper. The current OBNiSE architecture configuration is deployed in Figure 2.

**Figure 3:**
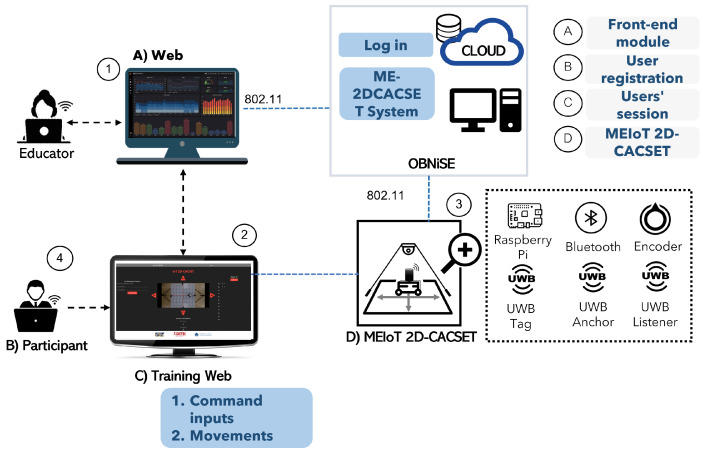
MEIoT 2D-CACSET system process implemented in the OBNiSE facilities.

The OBNiSE IoT architecture for MEIoT 2D-CACSET inspired by [33] contains a set of configurations and elements that allow interaction among them to communicate and transmit information through the devices at different system levels. Some of them are a web system, a training web part of the web system, the OBNiSE, and the MEIoT 2D-CACSET (see Figure 3). The users are divided into two categories, educators and participants. Only one participant at a time can modify the training website. Every element is described as follows:

1. Web System. It is stored in a cloud system in the OBNiSE facilities, which only educators can access. The web system performs the connection with the Raspberry Pi and displays the Mindstorms EV3 mobile robot coordinates in real time.
2. Training Web. This training web is part of the primary system with limitations for participants. In this, participants are only allowed to modify the coordinates of the LEGO Mindstorms EV3 mobile robot in Y−, Y+, X−, X+, and visualize the movements that the robot executes to reach them in real time. It is only allowed to display up to 10 movements of the robot at a time. The platform has a “clean” button, which helps if anyone wants to see other coordinates or delete them at any time, so the participants do not have to wait until the movements are reached.
3. MEIoT 2D-CACSET. It is an IoT device that integrates a Raspberry Pi for the robot control, a Bluetooth module, an encoder, an ultrawide band (UWB)-tag, UWB anchor, and UWB listener modules. The MEIoT 2D-CACSET establishes the communication between the training web and the LEGO Mindstorms EV3 mobile robot via Wi-Fi to capture the coordinates sent from the training website and then execute them in the robot.
4. Users. These are divided into two categories.
  - Educators. They have access to all the data and arrangements of the MEIoT 2D-CACSET system and have complete knowledge of handling the configurations. Likewise, they allow or deny access to registered participants in the system. Educators also can modify the dashboard, data, time of actualization, color, and other functionalities.
  - Participants. These are primarily students who are part of the mechatronics education course, interns, and social service students. They require registration on the platform to access the system and view the MEIoT 2D-CACSET. The participants have limited access and can only modify some parameters that the educator allows for a defined time.

## 5. MEIoT 2D-CACSET: Two-Dimensional Cartesian Coordinate System Educational Toolkit

**Figure 4:**
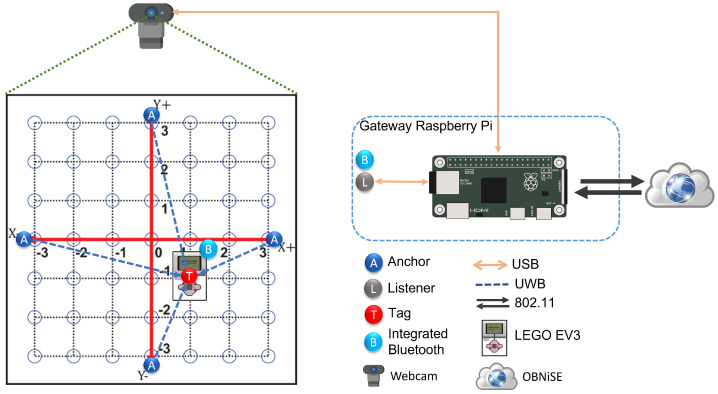
Educational toolkit MEIoT 2D-CACSET complete architecture.

The MEIoT 2D-CACSET is an educational toolkit that teaches the Cartesian Coordinate System (CCS) that applies the EMCF-LCM. Adding media that allows interaction between students and the toolkit introduces a virtual level into the 2D-CACSET toolkit. Earlier work [16] emphasizes the possibility of installing the Real-Time Location Systems (RTLS) technology in different mechatronics prototypes, such as mobile robots, drones, and manipulators; by taking advantage of this feature, students be able to use MEIoT 2D-CACSET. The proposed architecture for the MEIoT 2D-CACSET involves using 2D-CACSET, Mindstorms EV3 mobile robot as an interactive actuator (EV3IA), the OBNise framework, a Raspberry Pi, and a webcam, which is depicted in Figure 4.

**Figure 5:**
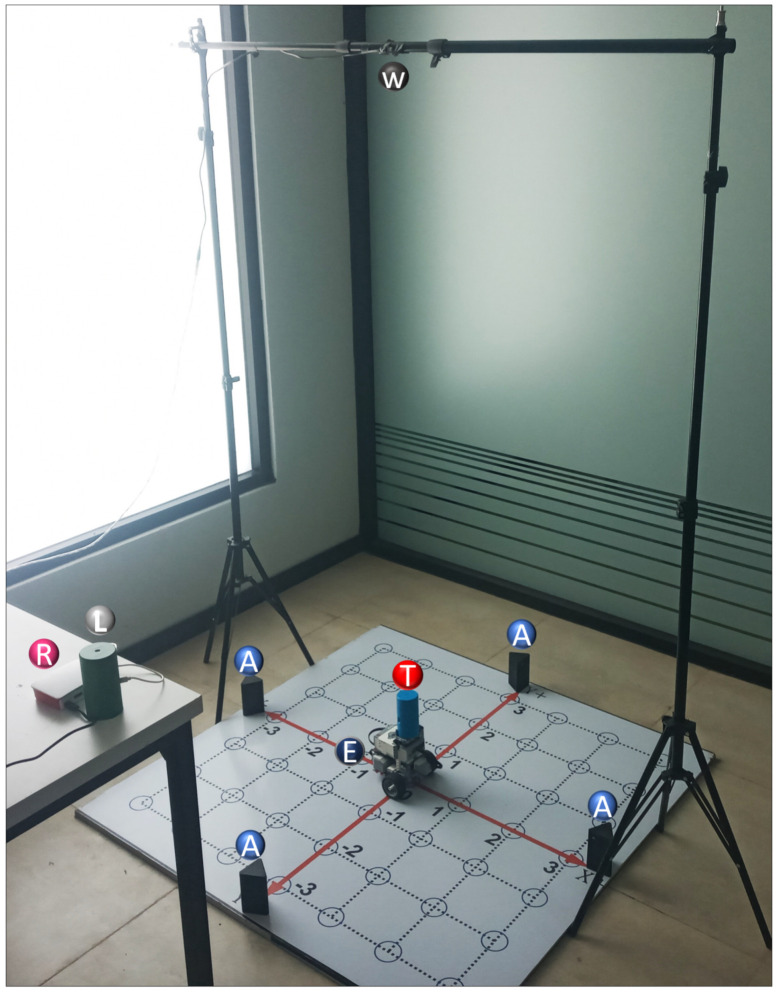
Hardware setup: anchor devices (A), tag device (T), EV3IA (E), listener device (L), Raspberry Pi (R), and webcam (W).

According to the proposed architecture depicted in Figure 4, the experimental setup in Figure 5 was executed at the Autonomous University of Zacatecas where there is a stable connection to the internet, a crucial for any IoT elements. The deployment consists of the 2D-CACSET located on the floor and a webcam positioned above it at the height of 1.70 m so that it has the complete workspace area view. A listener device is located near 2D-CACSET to listen for coordinates data. The webcam and listener device are connected to the Raspberry Pi via USB. The EV3IA is placed at the center of the workspace, which corresponds to the origin of CCS, waits for any commands, and at the same time reports positioning data to the Raspberry Pi listener device.

The following sections describe the elements that constitute the proposed architecture and critical features and the specific functions they perform.

### 5.1. Raspberry Pi Gateway

**Figure 6:**
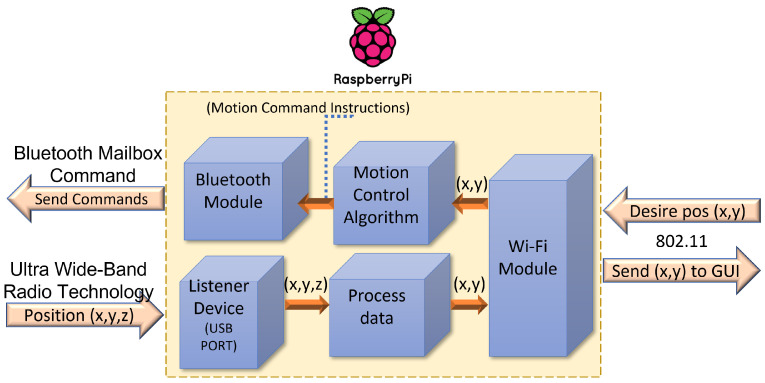
High diagram connection components of the Raspberry Pi.

Into IoT architecture, the gateway is responsible for collecting data from the edge devices through any proprietary/custom protocol embedded to be delivered through the Internet. The gateway device enables the interface towards the edge devices and, on the other side, acts as a user’s device for transmitting data toward the final destination. For this purpose, an SBC Raspberry Pi 4 B+ is employed, where several devices were connected, namely, a listener device, to collect data positioning from tags and a USB camera, and to establish visual contact with the workspace. The Raspberry Pi 4 B+ uses the Bluetooth module for connecting with the EV3IA and Wi-Fi network to uplink/downlink the information to/from the GUI. Figure 6 depicts a high-level diagram representing the components of the Raspberry Pi.

**Figure 7:**
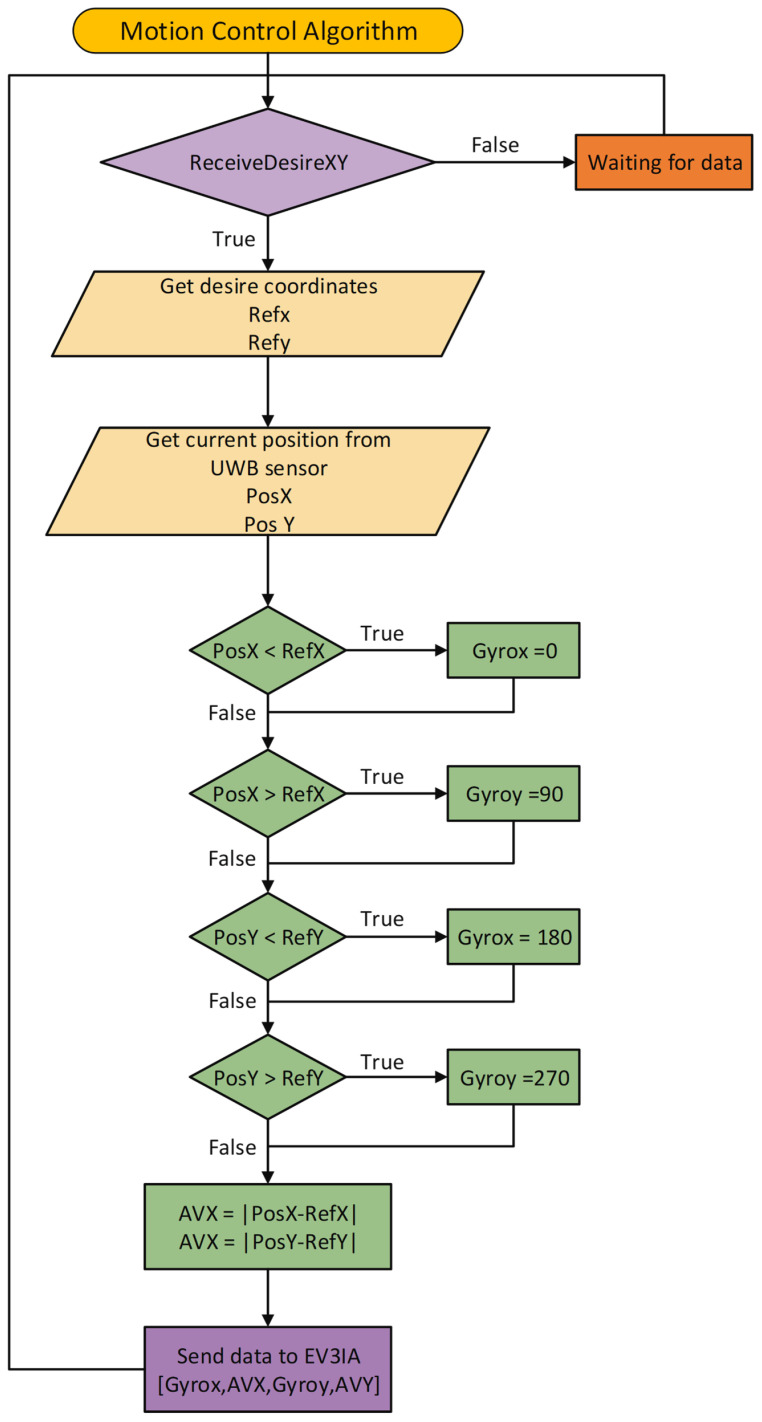
Motion control algorithm: the arguments of the functions is the desired position coordinates (x and y) introduced by the user from the GUI; the output is an array of instructions that the EV3 will decode and execute.

Sockets were used to communicate the Raspberry Pi with the GUI; promptly, a web socket with Node.js updates the status of the GUI. Moreover, a PHP script is used to receive data containing the ordered pair to which the user desires to move the interactive actuator, anddata are processed by the motion control algorithm, depicted in Figure 7, sending a series of commands to the EV3IA to conduct the motion commands.

**Figure 8:**
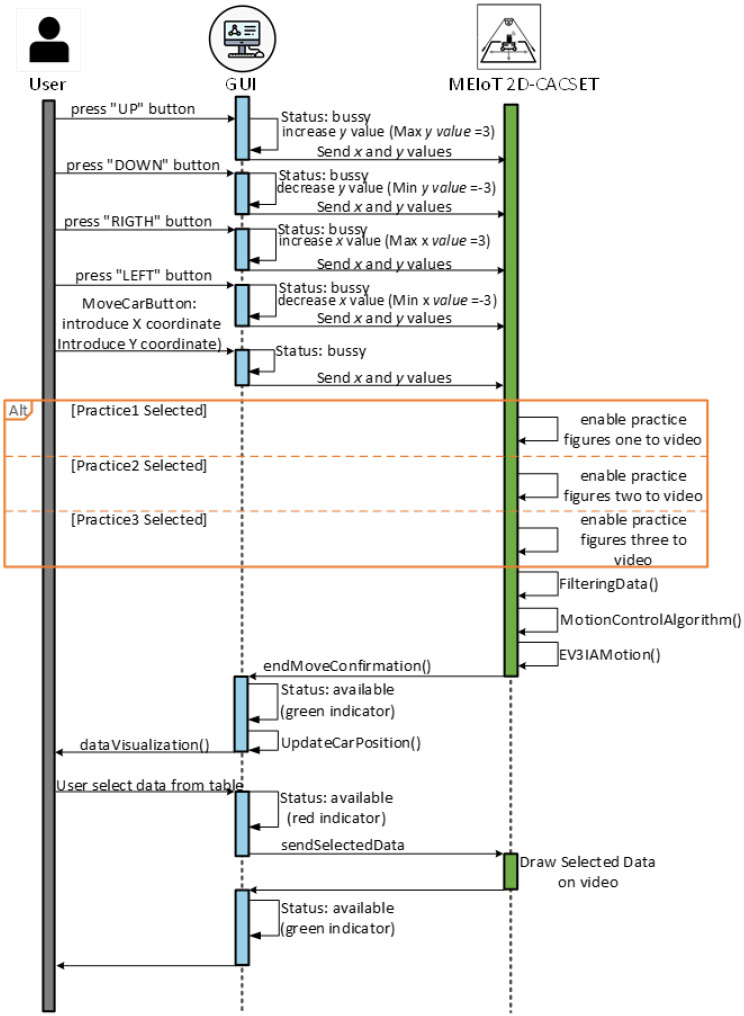
Sequential diagram connection between the user, the GUI, and the MEIoT2DCacset prototype.

In addition, the listener device accesses the positioning data of the EV3IA and Raspberry Pi process and prepares it to send it via Wi-Fi to the GUI. Raspberry enables a video streaming for the students to visualize the robot movements through GUI; for achieving this, an 8000 K pixel USB driver-free camera is employed, and the tasks must be executed asynchronously. While considering Raspberry Pi, it is a CPU-structured SBC, and hence the multi-threading concept is used. Our main thread performs the task of reading and decoding the data frame collected by the listening device. A video thread is created to process the record captured by the webcam to the GUI; one thread handles Bluetooth-related tasks, and so on. Last, our main thread is in charge of carrying out the trajectory planning of the EV3 interactive actuator. The diagram shown in the Figure 8 illustrates this distribution of the Raspberry Pi threads.

**Figure 9:**
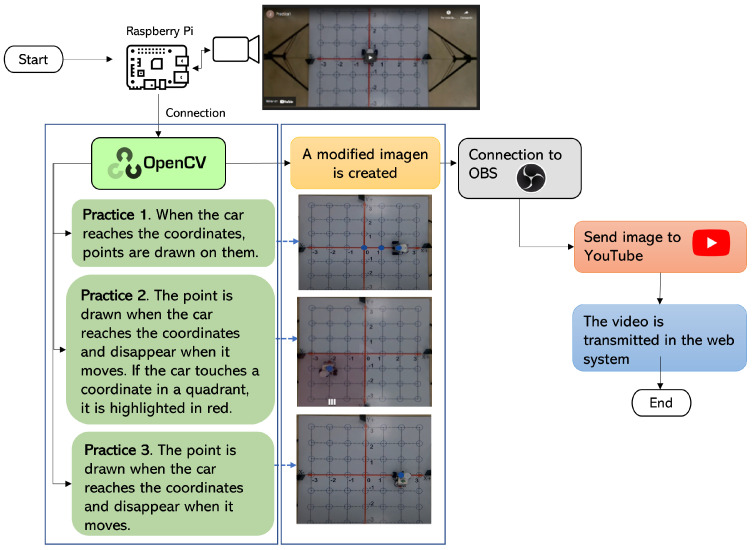
Connection between the Raspberry Pi and the GUI, using the OpenCV library and the OBS video streaming program.

To be able to equate the complete system to the methodology, different images need to be added to the video depending on the practice selected in the GUI (see Figure 9).

Different unit tests were carried out; each of them are described below:

- Raspberry Pi–web system unit test: The unitary test verifies the correct functionality and communication between the Raspberry Pi and the web system by exchanging data; the web sends commands to the Raspberry Pi to prove the arrival of the information and vice versa to control the status of the web (available, idle, or off).
- Camera–Raspberry Pi unit test: Connection tests were carried out between the camera and the Raspberry Pi to verify the correct operation and image visualization and set the image coordinates of the Cartesian plane, quality of the image, position, and the status of the camera.
- Raspberry PI–EV3IA unit test: The unitary test verifies the Bluetooth communication between Raspberry Pi and EV3IA by interchange mailboxes. To confirm the arrival of the data to the devices, a hyperterminal is implemented on Raspberry Pi using a serial port, and for the EV3IA, an SSH connection is conducted.
- OBS–Raspberry PI unit test: These tests verify the proper operation of practices using the OpenCV library, by adding the corresponding figures to the 2D board according to the selected practice. Moreover, this unitary test verifies the streaming video using OBS embedded in the web.

Following Figure 9, the camera must be configured in the Raspberry Pi to capture the video’s image; the configuration is performed using the OpenCV library and Python. When an image is ready using the OpenCV library, the adjustment must be made according to the practice selected in the GUI. The practice settings are as follows:

- Practice 1. The EV3IA, from its initial position, moves towards the coordinates that were sent through the system web. Once it passes through each of the coordinates in its path, it illuminates them until it reaches its destination. The points remain until the car stops.
- Practice 2. EV3IA leaves its initial position touring the Cartesian plane; when it passes any of the coordinates on its way, it draws a point that disappears once the EV3IA leaves the coordinates; if any of the coordinates are within a quadrant, this lights up in red, and the dialed number appears in white.
- Practice 3. The EV3IA moves from its initial position and draws points on the coordinates it visits on its way; once it continues moving, the point disappears and reappears at the new coordinate. Points stop being drawn when it reaches their final coordinates (see Figure 9).

A modified image is created according to the practice and presented in an external window; only one practice can be seen at once. The image of the practice that is running will be displayed. This image created in the previous step is sent to OBS (Open Broadcaster Software). OBS is free and open-source for video recording and live streaming. The connection can be easily made using Python. Once OBS has a stable connection with the OpenCV library and the Raspberry, the video is sent to YouTube. There is a connection from the web system to YouTube; then, the video can be streamed in real time on the web system so the practitioner can observe it. If the user selects another practice, the whole process is performed again to present the new setting of the chosen practice.

### 5.2. GUI MEIoT 2D-CACSET

**Figure 10:**
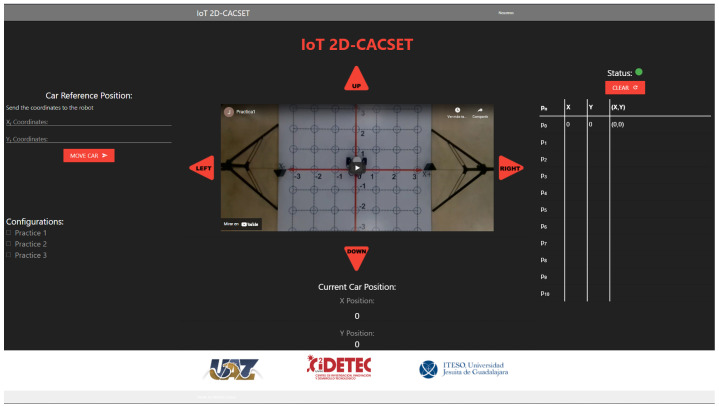
Graphical user interface: GUI MEIoT 2D-CACSET.

Several applications can be used for the visualization and manipulation of the data of an IoT application; for this, we develop the IoT-2D CACSET graphical user interface. The website’s navigation is intuitive and easy for the user to use, allowing students to interact with the 2D-CACSET through the EV3IA. The IoT-2D CACSET is depicted in Figure 10, and GUI elements are described below.

- EV3 Reference Position: The user can enter the desired coordinates to be reached by the EV3IA; by pressing the “MOVE CAR” button, the coordinate data are sent to the Raspberry Pi to request a movement of the EV3IA. Coordinates data entry are restricted according to the 2D-CACSET 2D workspace area.
- Configuration Check Box: The user can select between three interactive practices by selecting a checkbox; by selecting one of the checkboxes, interactive symbols and figures are enabled that appear in the video according to the chosen practice; this is described in depth in Section 6.
- Direction Buttons (Left/Up/Right/Down): The direction buttons allow the user to control the motion of EV3IA dynamically, and movements are performed unitary according to the pressed button, for example; if the EV3IA is at the origin of 2D-CACSET, then the down button is pressed and the EV3 should move to the position (0,−1).
- Video Streaming: The video is transmitted by using the OBS; this way, it enables the user to visualize the 2D-CACSET workspace remotely. Moreover, OpenCV is used to add figures according to the selected practice.
- Route Log Table: The table records all the movements made by the EV3IA. The user can select one of the registered coordinate positions, and a point will appear in the video transmission showing the chosen coordinate point.
- Current Car Position: Shows the current coordinates position of EV3IA.
- Status Indicator: The status indicator turns red, denoting a busy state, and green for an available state; these depend on the user’s interaction with the GUI. An example is when the user presses a button; at that moment, the status indicator will turn red, displaying that the system is attending to the request; other operations cannot be performed until the current one concludes.

All communication is via Wi-Fi. Additionally, as mentioned at the beginning of this section, this proposal is based on the original OBNiSE architecture. For a participant to make any changes to the system, they must follow the next steps.

**Figure 11:**
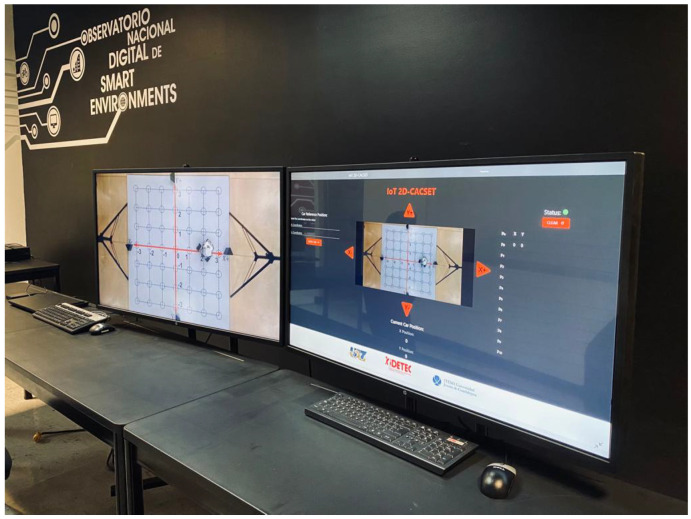
MEIoT 2D-CACSET system running at OBNiSE facilities.

1. Every participant must be previously registered on the training web and access using its username and password. Then, the server verifies the user to establish the connection with the EV3IA mobile robot (named robot in this section to simplify the explanation).
2. Once the participant is on the platform, they will be able to see the current state of the robot, the coordinates, and the visualization of these on the screen (see the right screen in Figure 11).
3. The participant will be able to modify the robot coordinates, which are configured in a range of (−3,3) in the *X* and *Y* axes. Once the coordinates have been entered, the participant presses the “Move Car” button.
4. Every coordinate or movement can be set individually on the camera by pressing the arrows around the video. The movements that can be made are UP (Y+), DOWN (Y−), LEFT (X−), and RIGHT (X+).
5. The coordinates are sent through the network to the server, and the server sends them to the MEIoT 2D-CACSET device.
6. The participant will then be able to visualize the robot’s movement on the platform in real time; the robot will stop once it has reached the coordinates.
7. The platform also contains a set of configurations. Every participant can select any of these configurations (practice 1, practice 2, and practice 3). Every practice performs a set of actions that users can see. The configurations can be selected one by one or more at a time.
8. Finally, the participant will also see the robot’s movements through the live camera, which is transmitted to one of the observatory’s screens (see the left screen in Figure 11).

Once the student’s time has expired, or they no longer want to continue modifying the robot’s coordinates, they must close their session to allow connection to the system from another participant.

### 5.3. LEGO Mindstoms EV3—Interactive Actuator (EV3IA)

**Figure 12:**
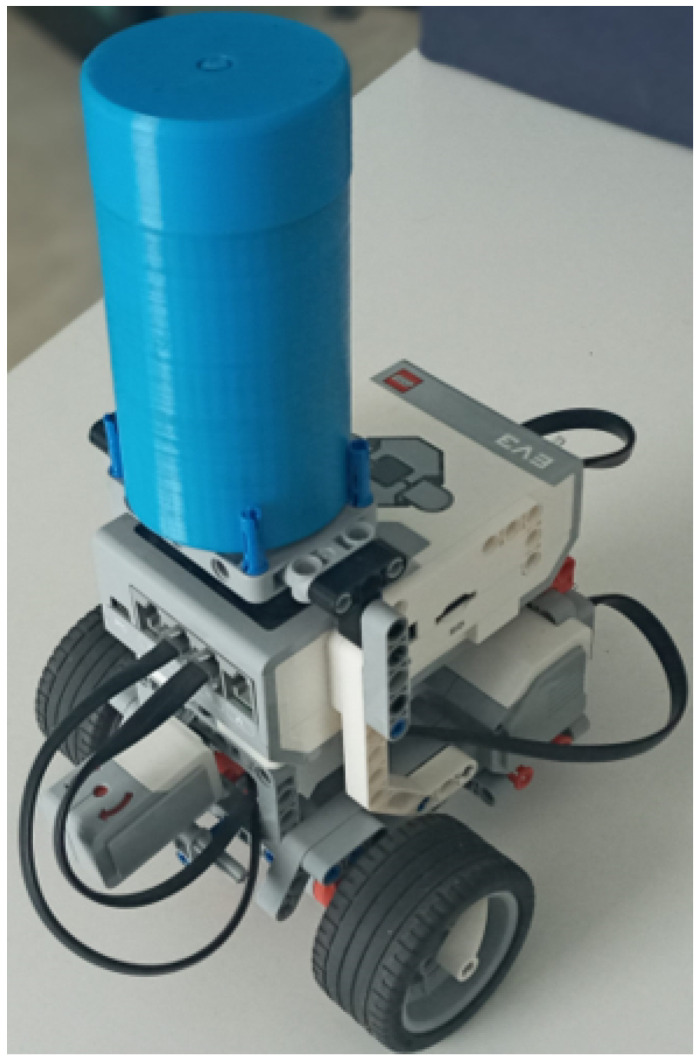
EV3 interactive actuator model equipped with a tag device and a gyroscope sensor.

For students to interact with the toolkit remotely, it is essential to provide the means that permit it. Robots are promising devices that can be programmed and optimized to conduct diverse tasks with high accuracy, offering high versatility for adapting sensors, actuators, and other peripheral devices [34]. Such is the case with the LEGO Mindstorm Robots, which is an educational platform for robots design and development, supported by the LEGO group. To date, LEGO has brought three generations of Mindstorm control for robots: the Mindstorms RCX (Robotic Command eXplorer), which contained a variety of LEGO parts and pieces as well as some customized motors and sensors; the Mindstorms NXT (The Next Generation), which includes motors, sensors, and a variety of parts and pieces used to create virtually any type of robot of anyone can imagine; and the third generation, known as Mindstorms EV3 (The Evolution), released on 1 September 2013, which has four input ports, four output ports, a mini USB port, a USB port, an SD card slot, a speaker, five buttons (center, right, left, up, down, and back button), backlight button with three colors (red, orange, green) and screen larger than that of NXT. It also has 64 times more memory than NXT and EV3 and is plug and play, created to be used as a part of the teaching activities of many subjects or disciplines in classes [35,36,37]. Taking advantage of these features, in this work, LEGO MINDSTORMS® EV3 is implemented in conjunction with a tag device and a gyroscope sensor, thus creating an interactive actuator; tag is placed in EV3, employing a variant of the building instructions for Robot Educator, as is shown in Figure 12.

**Figure 13:**
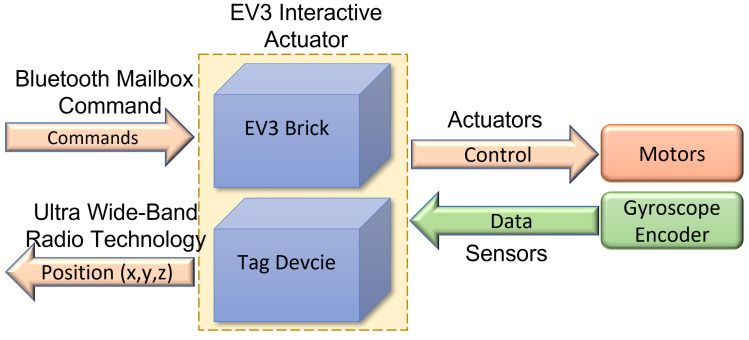
High-level EV3 components diagram using sensors and motors.

The EV3IA work as an edge device in the general OBNiSE IoT architecture and performs two main tasks: execute kinematics introduced by users via the GUI and notify its positioning data. Tasks run in parallel, assuming they are separate devices (EV3 and tag). To notify position, tag sends, via UWB technology, *X* and *Y* coordinates every 100 ms, and a listener device recollects this information; these data can be used for the GUI. For kinematics control, it is necessary to program several different interactive actions that the controller has to perform. LEGO Mindstorms EV3 is typically programmed using EV3 Classroom LEGO® Education, a standard LEGO icon-based software to create executable code for the EV3 containing the original LEGO firmware. EV3 supports Linux operating system and could be programmed using other languages such as C++, Java, EV3 basic, and Python, among others. Although it would mean modifying the firmware [36], employing those programming languages allows for performing more advanced activities such as [38], which might not be possible with the standard EV3 programming firmware. LEGO provides an EV3 MicroPython image that permits the programming of EV3 using MicroPython; in this approach, MicroPython programming language is used, including APIs and drivers that allow the user to control all the EV3 peripherals and obtain data from EV3 sensors, consequently allowing a more profound kinematics control. To receive commands, EV3 Bluetooth Messaging Service serves clients by processing incoming commands and sending replies; according to that documented in the EV3 Firmware Developer Kit [39], naturally, EV3 has been programmed to wait for Raspberry Pi clients. Figure 13 depicts a high-level diagram representing the components of the EV3IA and its sensors and motors.

**Figure 14:**
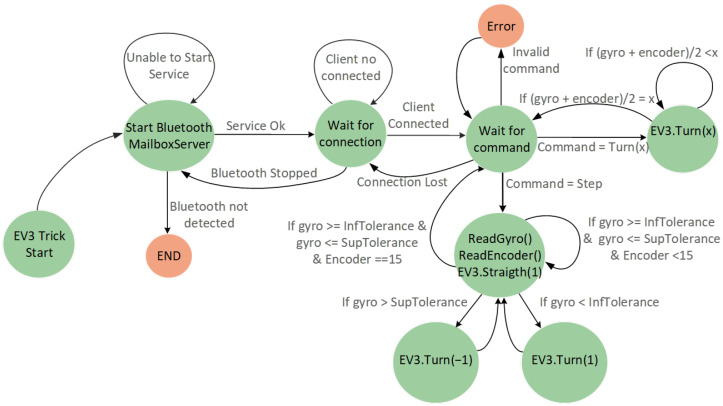
Machine state diagram that represents the EV3IAMotion() algorithm.

The motion embedded code, shown in Figure 14, was implemented in the EV3IA, using internal rotation sensors and a gyroscope to determine a travel distance and a specific turning angle. When a “Step” command is received, EV3IA performs a movement to the next point in CCS, and the “Turn (x)” command executes a turn according to the argument of the function; those motion commands perform under the workspace dimensions and spaces between points on the 2D board limitations.

## 6. Instructional Design for Mechatronic Concept Two-Dimensional Cartesian Coordinates Using the IoT 2D-CACSET

Since this research is the extension of a previous work presented in [16], we decided to keep the same learning objective: improving the mechatronic thinking within the educational phase framework using modern IoT technology from a remote location. Then, this paper focuses on virtual learning for the two-dimensional space concept using the MEIoT 2D-CACSET, including the three learning levels of the EMCF: virtual concrete, virtual graphic, and virtual abstract level. These are described below:

- Virtual Concrete Level: This level requires the student to experiment with virtual-world objects; therefore, the MEIoT 2D-CACSET represents a way to achieve this. The bidimensional CCS is a widely used way to locate objects. Consequently, it means the best choice to start with basic spatial concepts. For this purpose, the MEIoT 2D-CACSET includes a mobile robot equipped with a tag to report its current position concerning the board that represents the Cartesian plane. It is worth mentioning that even though the mobile robot is an actual artifact, it is considered a virtual object since the participant will be interacting through the GUI MEIoT 2D-CACSET from a remote location in a virtual manner. The participant can interact and command the movement of the mobile robot with the tag, which represents a point in the plane, through the control buttons “RIGHT”, “LEFT”, “UP”, and “DOWN”, which use instructions in colloquial language. It is essential to mention that the GUI MEIoT 2D-CACSET will graphically show the reached point cloud, using a series of interconnected filled circles as the mobile robot moves on the board.
- Virtual Graphic Level: In this second level, the participant must relate the skills acquired in the first level with symbolic elements. In this case, the participant can see all the circles representing the points that the mobile robot with the tag is reaching. Moreover, a CSV file containing the tag’s raw data position can be downloaded for analysis using Matlab to reinforce this level.
- Virtual Abstract Level: Abstract logical thinking is acquired at the stage of formal operations. Therefore, students can develop a mathematical understanding to enhance cognitive processes without relying on manipulating an object. For this purpose, the current *x* and *y* position values of the tag are displayed on the right side table of the GUI MEIoT 2D-CACSET video. Every time the tag reaches a new position, this is added to the table. Then, the participant can relate these values to the filled circles. Moreover, the mentioned CSV file in the previous level could be used for a better understanding of the variables.

Following the EMCF-LCM methodology, three virtual interactive practices are proposed. Each practice covers the three learning levels reviewed previously.

### 6.1. Practice 1: Cartesian Coordinate System

This practice will help the student understand the concept of coordinate axes. To achieve this, the complete MEIoT 2D-CACSET must work with a tag mounted on the mobile robot. The workspace is defined with a range of [−2,2] in the *X* and *Y* axes.

**Figure 15:**
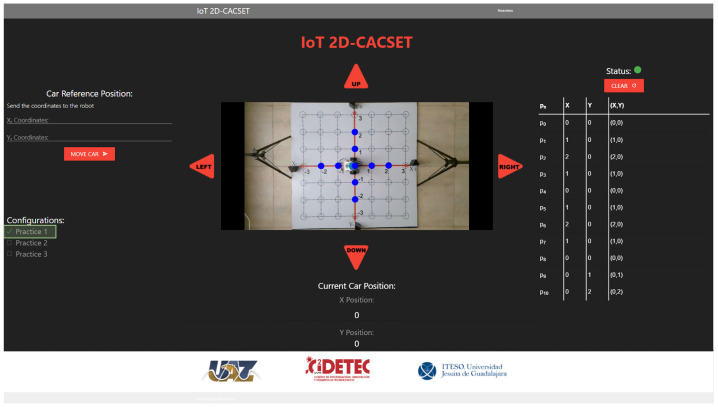
GUI MEIoT 2D-CACSET once the virtual concrete level has been performed by the participant.

**Figure 16:**
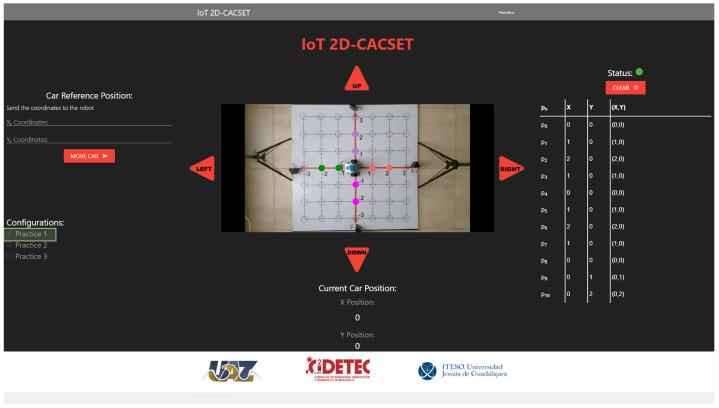
GUI MEIoT 2D-CACSET once the virtual graphic level has been performed by the participant. Note that origin, X+, X−, Y+, and Y+ axes are represented by the gray, red, green, purple and magenta filled circles, respectively.

**Figure 17:**
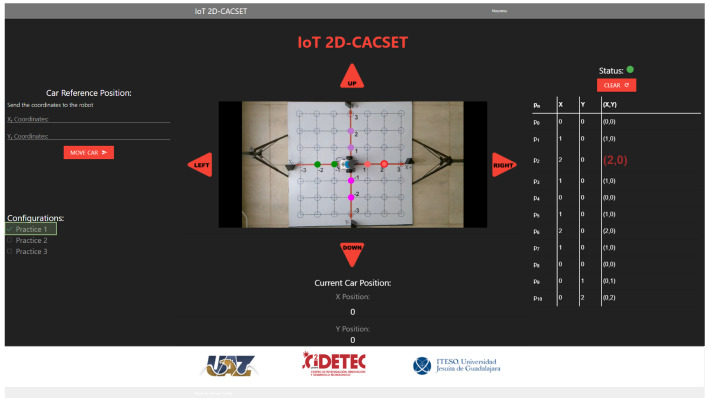
GUI MEIoT 2D-CACSET once the virtual abstract level has been performed by the participant. Note that the font size of point (2,0) is increased and its corresponding filled circle is highlighted.

● The set of instructions for the participants in the virtual concrete level are as follows:
  1. Move the mobile robot from its home position to the right direction by clicking once on the corresponding button; then, the robot starts moving one place in that direction and then stops. Click once more on the button in the right direction; then, the robot begins moving in one position and stops. Now, click once on the button in the left direction, then the robot starts moving in one place and stops. Finally, click once more on the button in the left direction, then the robot starts moving one position and then stops.
  2. Move the mobile robot from its home position to the left by clicking once on the corresponding button; then, the robot starts moving one position and stops. Click once more on the button in the left direction, then the robot starts moving one position and then stops. Now, click once on the button in the right direction; then, the robot begins moving in one position and then stops. Finally, click once more on the button in the right direction, then the robot starts moving one position and then stops.
  3. Move the mobile robot from its home position to the upward direction by clicking once on the corresponding button; then, the robot starts moving one position and then stop. Click once more on the button in the upward direction; then the robot starts moving one position and stops. Now, click once on the button in the downward direction, then the robot starts moving one position and then stop. Finally, click once more on the button in the downward direction, then the robot starts moving one position and then stop.
  4. Move the mobile robot from its home position to the downward direction by clicking once on the corresponding button; then, the robot starts moving one position and then stop. Click once more on the button in the downward direction, then the robot starts moving one position and then stop. Now, click once on the button in the upward direction, then the robot starts moving one position and then stop. Finally, click once more on the button in the upward direction, then the robot starts moving one position and then stop. Figure 15 shows the 2D board in the GUI once the concrete level instructions have been followed and performed.
● The set of instructions for the participants in the virtual graphic level participants are as follows:
  5. Make a color change of the filled circle from blue to gray by clicking on the circle where the mobile robot ends after the four steps of the specific level. This is the origin of the system *O*.
  6. Make a color change of the filled circle from blue to red by clicking on the circles where the robot passed when the first instruction of the concrete level was performed; exclude the home position. Then, assign to this movement the symbol X+.
  7. Make a color change of the filled circle from blue to green by clicking on the circles where the robot passed when the second instruction of the concrete level was performed; exclude the home position. Then, assign to this movement the symbol X−.
  8. Make a color change of the filled circle from blue to purple by clicking on the circles where the robot passed when the third instruction of the concrete level was performed; exclude the home position. Then, assign to this movement the symbol Y+.
  9. Make a color change of the filled circle from blue to magenta by clicking on the circles where the robot passed when the first instruction of the concrete level was performed; exclude the home position. Then, assign to this movement the symbol Y−. Finally, the participant obtained a plus shape draw (see Figure 16). The table containing the points reached by the robot can be seen on the right side of the GUI. The table contains the points reached by the robot.
● The set of instructions for the participants in the virtual abstract level participants are as follows:
  10. The plus shape is the representation of the 2D CCS. The horizontal line from the origin to the farthest point to the right and left are called the $\vec{OX+}$ and $\vec{OX-}$ axes, respectively. The complete horizontal line is known as *X* axis, containing the set of real numbers {−2,−1,0,1,2}. Moreover, the vertical line from the origin to the farthest point to the top and bottom are called the $\vec{OY+}$ and $\vec{OY-}$ axes, respectively. The complete vertical line is known as *Y* axis, containing the set of real numbers {−2,−1,0,1,2}. In addition, the intersection of the horizontal and vertical line is known as the origin, with representation in 2D coordinates (0,0); the first coordinate corresponds to the *X* axis and the second coordinate to the *Y* axis.
  11. Using the data in the GUI, build a table containing all the 2D coordinates for the $\vec{OX+}$, $\vec{OX-}$, $\vec{OY+}$, and $\vec{OY-}$ (see Table 1).
  12. We can extend this knowledge to assign a pair of coordinates to any filled circle in the MEIoT 2D-CACSET board to represent a point in the 2D CCS with (x,y) coordinates. If any (x,y) coordinate in the table is clicked, the font size will be increased, and the corresponding filled circle on the 2D board will be highlighted.
  13. Finally, click on the point (2,0) in the Table of the GUI. It can be noted that the font size of the 2D coordinate is increased, and the corresponding filled circle on the 2D board is highlighted. We can extend this knowledge to any pair of coordinates in the GUI table (see Figure 17).

**Figure 18:**
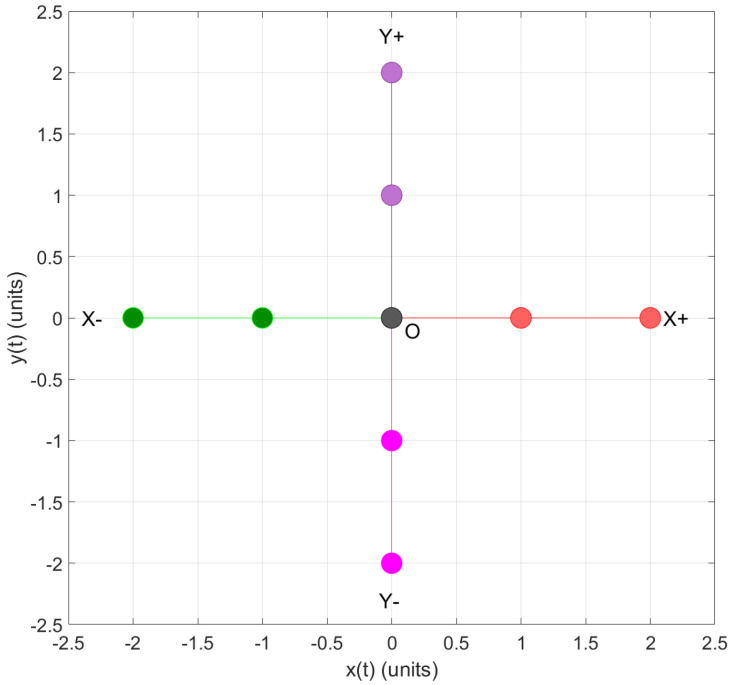
2D graph of tag position in Practice 1; plotted using Matlab.

**Figure 19:**
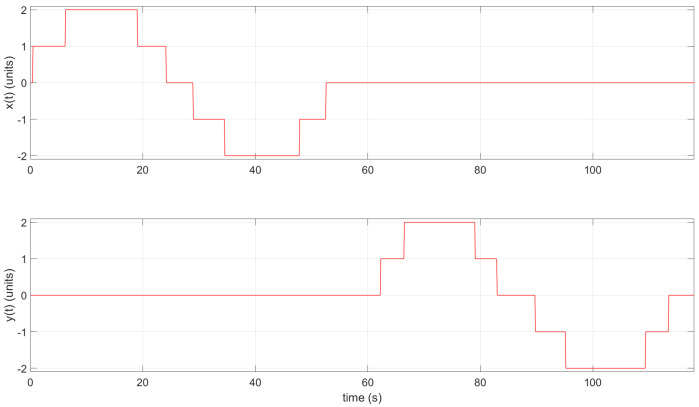
The *x* and *y* tag position with respect to time *t* in Practice 1; plotted using Matlab.

Moreover, when necessary, the instructor and/or participant can download the CSV file and open it using Matlab. This could be useful when working in an offline scenario. The collected data from the tag representing the position values with *x* and *y* values can be plotted in Matlab (see Figure 18 and Figure 19).

After completing practice, participants learned how a point moves along the positive and negative *X* and *Y* axes and how the 2D CCS is built. A video of Practice 1 can be seen in https://www.youtube.com/watch?v=pPzDQoNOXQY (accessed on 8 June 2022).

### 6.2. Practice 2: Quadrants

Now that we know the 2D CCS, this second practice will help the student understand the concept of the four regions of a 2D plane, called quadrants.

● The set of instructions for the participants in the virtual concrete level:
  1. Move the mobile robot from its home position to the right direction by clicking twice on the corresponding button; then, the robot starts moving two positions and then stops. Now, click twice on the button in the upward direction, then the robot starts moving in two positions and then stop. It can be noted that the mobile robot reached position (2,2).
  2. Move the mobile robot from its current position to the left by clicking four times the corresponding button; then, the robot starts moving in four positions and stops. It can be noted that the mobile robot reached position (−2,2).
  3. Move the mobile robot from its current position to the downward direction by clicking four times on the corresponding button; then, the robot starts moving four positions and then stop. It can be noted that the mobile robot reached position (−2,−2).
  4. Move the mobile robot from its current position to the right direction by clicking four times the corresponding button; then, the robot starts moving in four positions and then stops. It can be noted that the mobile robot reached position (2,−2).
  5. Finally, to return the mobile robot to the home position, click twice on the up button; then, the robot starts moving in two positions and then stops. Then, click twice on the button in the left direction, and the robot starts moving in two positions and then stops. It can be noted that the mobile robot reached position (0,0).
● The set of instructions for the participants in the virtual graphic level:
  6. Insert a purple square by clicking on the endpoint of instruction 1 of the concrete virtual level, the purple point in the position (2,2). Then, assign to this area the Roman number I.
  7. Insert a green square by clicking on the endpoint of instruction 2 of the concrete virtual level, the green point in the position (−2,2). Then, assign to this area the Roman number II.
  8. Insert a magenta square by clicking on the endpoint of instruction 3 of the concrete virtual level, the magenta point in the position (−2,−2). Then, assign to this area the Roman number III.
  9. Insert a red square by clicking on the endpoint of instruction 4 of the concrete virtual level, the red point in the position (2,−2). Then, assign to this area the Roman number IV.
● The set of instructions for the participants in the virtual abstract level:
  10. The first quadrant of the 2D plane, called quadrant I, is the region labeled with the Roman number I. It can be noted that the (x,y) coordinates in this region are (+,+), respectively.
  11. The second quadrant of the 2D plane, called quadrant II, is the region labeled with the Roman number II. It can be noted that the (x,y) coordinates in this region are (−,+), respectively.
  12. The third quadrant of the 2D plane, called quadrant III, is the region labeled with the Roman number III. It can be noted that the (x,y) coordinates in this region are (−,−), respectively.
  13. The fourth quadrant of the 2D plane, called quadrant IV, is the region labeled with the Roman number II. It can be noted that the (x,y) coordinates in this region are (+,−), respectively.
  14. Using the data in the GUI, build a table containing all the reached 2D coordinates corresponding to quadrants I, II, III, and IV (see Table 2).

At the end of this practice, the student learned that the 2D plane is divided by four regions called quadrants. The enumeration of these quadrants is made counterclockwise, starting from quadrant I to IV. The complete Practice 2 can be seen in the following video: https://www.youtube.com/watch?v=POr3KGLLBO8&t=13s (accessed on 8 June 2022).

**Figure 20:**
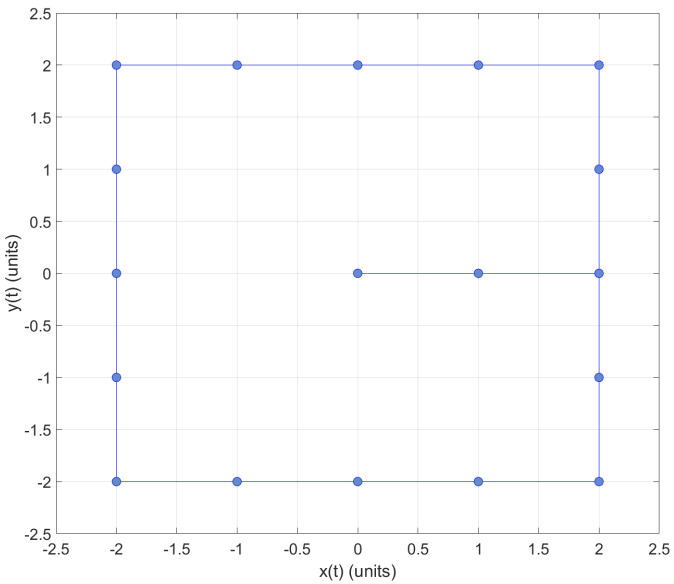
2D graph of tag position in Practice 2; plotted using Matlab.

**Figure 21:**
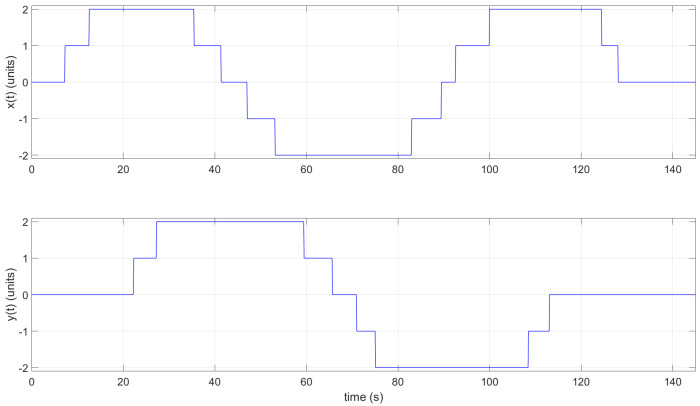
*x* and *y* tag position with respect to time *t* in Practice 2; plotted using Matlab.

Moreover, when necessary, the instructor and/or participant can download the CSV file and open it using Matlab. This could be useful when working in an offline scenario. The collected data from the tag representing the position values with *x* and *y* values can be plotted in Matlab (see Figure 20 and Figure 21).

### 6.3. Practice 3: Point in Cartesian Coordinate System

Now that we know the 2D CCS and its quadrants, this third practice will help the student understand the concept of locating a point in the plane.

● The set of instructions for the participants in the virtual concrete level:
  1. In the car reference position section of the GUI, introduce the reference position: $x_{r}=2$ and $y_{r}=2$ coordinates. Now, click the MOVE CAR button, then the mobile robot starts moving to the reference position and then stops. It can be noted that the mobile robot reached position (2,2).
● The set of instructions for the participants in the virtual graphic level:
  2. Make a color change of the filled circle from blue to purple by clicking on the circle where the mobile robot ends after the virtual concrete level is performed.
● The set of instructions for the participants in the virtual abstract level:
  3. The circle you filled with purple color can be seen as very small, almost dimensionless. It is perceptible by a contrast of color and it is called a point. The point coordinates are defined by an ordered pair with two numbers written together in parentheses. The *x* and *y* coordinates are commonly named abscissa and ordinate, respectively. The coordinates of point *P* are (2,2). Click on the point (2,2) in the table of the GUI. It can be noted that the font size of the 2D coordinate is increased, and the corresponding filled circle on the 2D board is highlighted. Table 3 shows the collected data from the tag, representing the position value of (x,y) coordinates.

At the end of this practice, the student learned the concept of a two-dimensional point. The complete Practice 3 can be seen in the following video: https://www.youtube.com/watch?v=TDPNNkp5YRQ (accessed on 8 June 2022).

**Figure 22:**
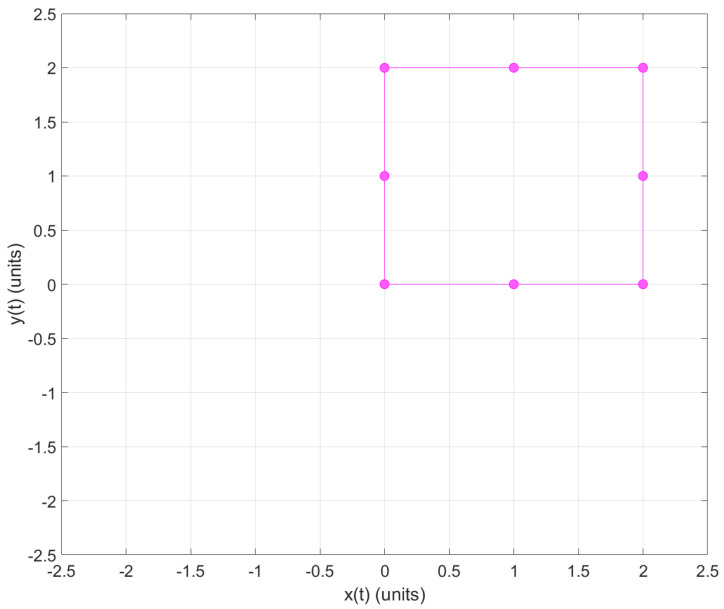
2D graph of tag position in Practice 3; plotted using Matlab.

**Figure 23:**
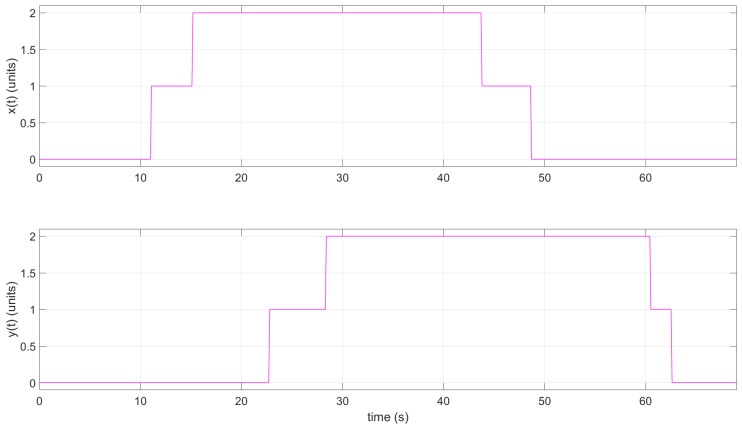
2D graph of tag position in Practice 3; plotted using Matlab.

Moreover, when necessary, the instructor and/or participant can download the CSV file and open it using Matlab. This could be useful when working in an offline scenario. The collected data from the tag representing the position values with *x* and *y* values can be plotted in Matlab (see Figure 22 and Figure 23).

## 7. Discussion

The MEIoT 2D-CACSET based on the OBNiSE architecture provides the necessary IoT capabilities. It enables the evolution from a face-to-face educational toolkit to an interactive remote hands-on learning lab focused on CCS learning. Moreover, the addition of the LEGO EV3 allows the system to have an interactive actuator (EV3IA) to carry out the practices and conduct the desired movements. This is thanks to the developed motion algorithm, which uses the data collected by the following sensors: gyroscope, encoders, and UWB sensor, to generate kinematic movements according to a reference position. Moreover, in [22], the latency analysis is performed for an IoT remote FPGA laboratory; it is critical to have low latency in FPGA’s real-time processing so that the data are not misinterpreted. Results show a latency of 0.192 s; for this work, the latency obtained in [22] for the functionality of the MEIoT 2D-CACSET system is taken as a reference, assuming that this is a system with slow dynamics, which uses the Raspberry Pi as an intermediary between the instructions provided from the GUI and the EV3 robot and that it can have a latency time no greater than 0.2 s, considering the maximum response time of the server. The results show an acceptable accuracy for this application. Although the main goal was achieved, there exist elements that can improve the overall performance of the toolkit. One of them is to improve the accuracy of EV3IA motion by adding sensors with better characteristics; LEGO supports standard protocols such as I2C that we can leverage to integrate third-party sensors. A well-known sensor is the gyroscope/accelerometer MP6050, which offers better characteristics (scale ranges of ±250°/s, ±500°/s, ±1000°/s, and ±2000°/s, sample rate 8 kHz) compared with the native LEGO gyroscope (440°/s, sample rate 1 kHz).

It is worth mentioning that the current system can be adapted to use other mobile robots according to the resources and tools of a particular university. Moreover, other alternatives to mobile robots can be explored, for example, using visual and/or auditory indicators. Additionally, OBNiSE provides the possibility to scale the original scope, integrating technologies such as machine learning, computer vision, and network management, among others [40]. Taking advantage of these features, we can employ the webcam as an image sensor to develop a motion control based on computer vision, as has been proved in other works [34,41]. Multi-sensor data fusion can be applied due to all these sensors involved in the toolkit to reduce uncertainty and increase information about the state of the system [42]. Fusing the data of the EV3IA, UWB, and image (webcam) sensors and then employing a stochastic method to improve the position estimation of the actuator, such as the Kalman filter, thus, using a proper kinematics control, has been proved in [43].

Moreover, the architecture of the MEIoT 2D-CACSET opens the opportunity of applying data fusion either centrally, where a device receives all the raw information from the sensors to perform the data fusion, or as a distributed structure, where the process is divided between the elements to perform local estimations. Then, a central filter performs the data fusion to provide much more accuracy overall [43,44].

On the other hand, the educational methodology can help to stimulate active learning as well as create multiple learning models in different areas of knowledge [45]; compared with other works reviewed in Section 2, the virtual EMCF becomes an important differentiator. The virtual EMCF guidelines develop spatial thinking through virtual-concrete, virtual-graphic, and virtual-abstract levels in the student and ensure the appropriation of mechatronic concepts through instructional practices; in this case, oriented to the learning of CCS. The explored concepts in this work are the essential construction elements of the CCS: origin, axes, quadrants, and point coordinates. However, the proposed educational technology allows exploring and expanding its scope to concepts such as distance, angles, polar coordinate systems, and even a 3D coordinate system.

Finally, the MEIoT 2D-CACSET can be fully applicable to teaching practice in the future, whether in remote or face-to-face education. With this development, we believe this educational toolkit will find application in a broader approach to engineering education, help teachers and students in the teaching–learning process, and subsequently increase their performance through active learning. Educators must take advantage of new educational technologies so that students stop being mere information receivers and become agents who apply knowledge; this development is proof of this.

## 8. Conclusions

The adaptation and development from an educational kit to a smart educational kit that takes advantage of the IoT technology has been presented in this work. It involves a web application, the OBNiSE architecture, a Raspberry Pi, an EV3 LEGO robot, a webcam, and the 2D-CACSET toolkit. Our complete IoT setup solution represents the first step towards the virtualization of the EMCF-LCM to enable the students to construct knowledge from a remote location. It is worthwhile to mention that IoT technologies took a crucial role in this new emerging learning process and that the complete IoT setup solution can be replicated to be used in more educational kits devoted only to face-to-face learning. We can successfully transform a physical laboratory into an online remote laboratory and extend the education methodology to "virtual" levels. The MEIoT 2D-CACSET provides the advantage of remotely controlling and monitoring a mobile robot. Then, it is possible to progressively expand the students’ learning methods and topics including more elaborate experiments, such as path planning tasks, trajectory tracking control, and multi-agent formation, among others.

## Figures and Tables

**Table 1:** Positions of the mobile robot in Practice 1.

$\vec{\mathrm{OX}+}$	$\vec{\mathrm{OX}-}$	$\vec{\mathrm{OY}+}$	$\vec{\mathrm{OY}-}$
(x,y)	(x,y)	(x,y)	(x,y)
(0,0)	(0,0)	(0,0)	(0,0)
(1,0)	(−1,0)	(0,1)	(0,−1)
(2,0)	(−2,0)	(0,2)	(0,−2)
(1,0)	(−1,0)	(0,1)	(0,−1)
(0,0)	(0,0)	(0,0)	(0,0)

**Table 2:** Positioning collected data in Practice 2.

Quadrant I	Quadrant II	Quadrant III	Quadrant IV
(x,y)	(x,y)	(x,y)	(x,y)
(2,1)	(−1,2)	(−2,−1)	(1,−2)
(2,2)	(−2,2)	(−2,−2)	(2,−2)
(1,2)	(−2,1)	(−1,−2)	(2,−1)

**Table 3:** Positioning collected data in Practice 3.

(x,y)
(2,2)

## Data Availability

Not applicable.

## Abbreviations

The following abbreviations are used in this manuscript:

2D-CACSET	Two-Dimensional Cartesian Coordinate System Educational Toolkit
IoT	Internet of Things
CCS	Cartesian Coordinate System
COVID-19	Coronavirus Disease 19
CSV	Comma Separated Values
RTLS	DWM Real-Time Location Systems
DWM	Decawave Module
EMCF	Educational Mechatronics Conceptual Framework
EMCF-LCM	EMCF Learning Construction Methodology
GUI	Graphical User Interface
RF	Radio Frequency
RFID	Radio Frequency Identification
RTLS	Real-Time Location Systems
SPI	Serial Peripheral Interface
STEM	Science, Technology, Engineering, and Mathematics
TDoA	Time Differential on Arrival
TWR	Two-Way Ranging
UART	Universal Asynchronous Receiver–Transmitter
UWB	Ultrawide Band
VR	Virtual Reality
WAN	Wide Area Network
Wi-Fi	Wireless Fidelity
OBS	Open Broadcaster Software
RCX	Robotic Command eXplorer
NXT	The Next Generation
EV3	The Evolution
EV3IA	EV3 Interactive Actuator
HOL	Hands-On Learning
ME	Educational Mechatronics (for its acronym in Spanish)
MEIoT	Educational Mechatronics and Internet of Things
MEIoT-2D-CACSET	Educational Mechatronics and Internet of Things 2D-CACSET
